# Network and Pathway Analysis of Toxicogenomics Data

**DOI:** 10.3389/fgene.2018.00484

**Published:** 2018-10-22

**Authors:** Gal Barel, Ralf Herwig

**Affiliations:** Department Computational Molecular Biology, Max Planck Institute for Molecular Genetics, Berlin, Germany

**Keywords:** network analysis, protein–protein interaction network, pathways, drug toxicity, toxicogenomics, transcriptomics, anthracyclines

## Abstract

Toxicogenomics is the study of the molecular effects of chemical, biological and physical agents in biological systems, with the aim of elucidating toxicological mechanisms, building predictive models and improving diagnostics. The vast majority of toxicogenomics data has been generated at the transcriptome level, including RNA-seq and microarrays, and large quantities of drug-treatment data have been made publicly available through databases and repositories. Besides the identification of differentially expressed genes (DEGs) from case-control studies or drug treatment time series studies, bioinformatics methods have emerged that infer gene expression data at the molecular network and pathway level in order to reveal mechanistic information. In this work we describe different resources and tools that have been developed by us and others that relate gene expression measurements with known pathway information such as over-representation and gene set enrichment analyses. Furthermore, we highlight approaches that integrate gene expression data with molecular interaction networks in order to derive network modules related to drug toxicity. We describe the two main parts of the approach, i.e., the construction of a suitable molecular interaction network as well as the conduction of network propagation of the experimental data through the interaction network. In all cases we apply methods and tools to publicly available rat *in vivo* data on anthracyclines, an important class of anti-cancer drugs that are known to induce severe cardiotoxicity in patients. We report the results and functional implications achieved for four anthracyclines (doxorubicin, epirubicin, idarubicin, and daunorubicin) and compare the information content inherent in the different computational approaches.

## Introduction

To thoroughly study the mechanisms behind drug induced toxicity a robust analysis by means of computational methods is crucial (Liebler and Guengerich, 2005). Understanding the influence of the compounds on different biological processes is complex and requires sophisticated interpretations of the data. In the field of toxicogenomics transcriptome data, that were collected upon drug treatment and that reflect gene expression levels in response to it, is in the focus of the analysis. Various studies, both *in vitro* and *in vivo*, focusing on different compounds and organs, have been already carried out (Hartung, 2009). Most of the studies were based on microarray technology (Mei et al., 2010), even though newer technologies, such as high-throughput sequencing (RNA-seq), are already in use in other research areas. Such transcriptomic profiles have previously been used for predicting toxic drug effects (Gusenleitner et al., 2014; Kohonen and Parkkinen, 2017; Nystrom-Persson et al., 2017; Rueda-Zarate et al., 2017), but further analysis for identifying the functional and molecular mechanisms behind the toxic effects is still much needed.

Here, we describe different approaches for the analysis of toxicogenomics data at the molecular network and pathway levels. We use publicly available data from microarray experiments and perform a differential expression analysis in order to identify the genes that are up- or down-regulated due to the administration of the drug (DEGs: differentially expressed genes). Suggested methods and tools include (i) over-representation analysis, (ii) gene set enrichment (pathway) analysis, and (iii) network propagation. All methods are complementary and deliver different and complementary mechanistic views on drug action and drug effects derived from the underlying gene expression data. Over-representation analysis provides a first impression on which pathways and biological functions are involved in the cell’s response to the drug. This kind of analysis is typically done with statistical tests that evaluate the list of DEGs interrogating pre-defined gene sets that represent pathways or Gene Ontology (GO) functions. Gene set enrichment (pathway) analysis is a complementary approach in the sense that not only DEGs are investigated but rather the entire gene expression response. This ensures that not only those pathways appear interesting that agglomerate many DEGs but also those that agglomerate subtle but consistent transcriptome changes of many of their members (not necessarily DEGs). A third, more unsupervised approach is network propagation, a mathematical concept that traces the effects of perturbations (e.g., gene expression changes) simultaneously across a molecular network according to a specified rule. It assumes that a perturbation in a certain gene is not only affecting that particular gene but rather its entire network neighborhood. The signal induced by a perturbation is then propagated to the neighbors and the neighbors of the neighbors until a steady-state (or convergence state) is achieved. The result of the network propagation is a final state in which each node is assigned a final weight which can be used to identify highly affected nodes as well as specific interconnected parts of the network (network modules) that are mostly affected by the induced perturbations.

A previous effort to infer functional effects of drug treatments from gene expression data was done on a pathway level in the work of Hardt et al. (2016). They assembled the ToxDb database, which contains gene expression data for more than 400 drugs and 2000 pathway concepts. This includes the association of drugs with specific molecular pathways, which can indicate to which mechanisms of action lead to toxicity. Here, we make use of this resource and the proposed implementation to extract pathways that are relevant for the toxic effect of different drugs. Additionally, we apply the same scoring scheme for measuring gene and pathway responses from gene expression data and enact a network analysis in order to identify functional modules that can also be associated with the toxic effect (see Methods).

Biological interactions are often described using molecular interaction networks (Barabasi and Oltvai, 2004), where each node represents a biological player, i.e., gene or protein, and each edge describes an interaction between a pair of nodes. Analyzing these networks can help to better elucidate the functional mechanisms that are being studied (McGillivray et al., 2018). There are numerous types of biological interaction networks (Vidal et al., 2011), as they can be based on different types of interactions and represent various biological actions. They vary between depicting gene regulatory interactions, viral-host interactions, metabolic reactions, protein–protein interactions, and more. Many of these interactions have already been made publically available through various specific databases, such as Reactome (Matthews et al., 2009), PID (Schaefer et al., 2009), KEGG (Kanehisa et al., 2012), and many others. Furthermore, there have been several attempts to combine and integrate different resources into one meta-resource, such as the work by Martha et al. (2011), IntNetDB (Xia et al., 2006), and ConsensusPathDB. In this work we make use of ConsensusPathDB (Kamburov et al., 2009), which currently integrates more than 600,000 interactions of different types which are collected from 32 public resources (Kamburov et al., 2013). Furthermore, we restrict our analysis to protein–protein interactions which are generally based on various experimental technologies (Walhout and Vidal, 2001).

One possible use of biological networks is for the identification of smaller subnetworks (subgraphs within the network), also referred to as modules, which depict an area that is more relevant for a specific biological function (Gustafsson et al., 2014). By integrating experimental data with interaction networks we can compute subnetworks that better represent the biological mechanisms which lead to a specific phenotype. There are several existing algorithms for module detection in biological networks. For a comprehensive overview of the different methods, see the recent review by Cowen et al. (2017). Many of these algorithms are based on a random walk process, where the weights of the nodes are propagated through the network, until a steady state is reached. The weighing of the nodes is dependent on the specific context and can be extracted for example from gene expression values or genetic mutation data. In this work we make use of the HotNet2 algorithm (Leiserson et al., 2015) that was originally developed for identifying subnetworks that result from somatic mutations. We apply the algorithm to toxicogenomics data and identify the most significant subnetworks for a drug treatment based on the gene expression response scoring.

We exemplify our approach on anthracycline drugs. Anthracyclines are a family of drugs that induce cardiotoxicity upon cancer treatment, and their use can result in cardiomyopathy and heart failure in many cases after a long period of time after treatment (Geisberg and Sawyer, 2010). These compounds are vastly used as chemotherapy agents, and have been shown to be extremely effective, but also to cause a major morbidity in cancer patients due to their toxic effects (Lenneman and Sawyer, 2016). Every exposure to anthracyclines carries some risk of resulting in cardiac dysfunction. The symptoms could present early on as well as at later times, in up to 23% of the patients (Steinherz et al., 1991). Although it is known that anthracyclines disrupt the synthesis of DNA and RNA, mainly by inhibiting topoisomerase II (Geisberg and Sawyer, 2010) and that they lead to mitochondrial dysfunction (McGowan et al., 2017), the mechanisms that cause the cardiotoxic effects still remain largely unclear (Truong et al., 2014). Previous studies have tried to elucidate this problem, however, there is still need for further investigation so that detection and prevention could be improved (Raschi et al., 2010). We focus our analysis on the four most widely used compounds: daunorubicin, doxorubicin, epirubicin, and idarubicin. In addition, we compare the results to other chemotherapy agents from different drug families, which are also known to cause cardiotoxic effects.

## Materials

### DrugMatrix

The toxicogenomics DrugMatrix (Ganter et al., 2005) database includes gene expression experiments from different rat tissue types at different time points and drug dosages. Data were downloaded via the diXa data collection (Hendrickx et al., 2015) that is available at http://wwwdev.ebi.ac.uk/fg/dixa/index.html. This data collection includes toxicogenomics profiles for 372 different compounds that were collected using the Affymetrix whole genome 230 2.0 rat GeneChip array. Data are available for heart, kidney, liver and muscle tissues, as well as for hepatocytes. The experiments were conducted for up to five times (after 0.25, 1, 3, 5 and 7 days) with only one dose concentration. In some cases, more than one dose was tested, and in others only one or two time points were measured.

### Anthracycline Expression Data

The analysis was focused on four different anthracyclines compounds: daunorubicin (CHEMBL178), doxorubicin (CHEMBL53463), idarubicin (CHEMBL1117), and 4-epidoxorubicin (CHEMBL1237042). We downloaded the CEL files from the DrugMatrix database via the diXa data collection for these compounds in heart tissue. A full description of the treatments is given by Table 1. Daunorubicin is the only compound for which data are available at two doses, a higher “toxic” dose and a lower “pharmacological” dose (Ganter et al., 2005). Thus, for the analysis of daunorubicin we used only the higher dose.

**Table 1:** Anthracyclines drug treatment experiments from the DrugMatrix database.

Drug	Time points (days)	Dosage (mg/kg)
Daunorubicin	1, 3, and 5	2/3.25
Doxorubicin	1, 3, and 5	3
Idarubicin	1, 3, and 5	0.625
4-Epidoxorubicin	1, 3, and 5	2.7

### Expression Data From Other Cardiotoxic Drugs

In order to evaluate the molecular effects that were identified in this work for the anthracyclines we also applied our workflow to three other chemotherapy agents that are known to induce cardiotoxicities (Truong et al., 2014). Out of 41 drugs that are mentioned in the review by Truong et al., only these three had data available in the DrugMatrix database. Cyclophosphamide (CHEMBL88) and ifosfamide (CHEMBL1024) are both alkylating agents, and imatinib (CHEMBL941) is from a family of small-molecule targeted therapy drugs. We downloaded the CEL files from the DrugMatrix database for these compounds in heart tissue. A full description of the treatments is given by Table 2. Data for imatinib were available in two different doses, and so we applied our analysis for the higher dose only.

**Table 2:** Other chemotherapy drugs and their experiments information from the DrugMatrix database.

Drug	Time points (days)	Dosage (mg/kg)
Cyclophosphamide	3 and 5	25
Ifosfamide	3 and 5	143
Imatinib	1, 3, and 5	15/150

### ConsensusPathDB – A Molecular Interaction Network Resource

ConsensusPathDB (Kamburov et al., 2009) is a meta-database for molecular interactions and pathways that currently integrates 32 public resources (Kamburov et al., 2013) and is composed of more than 600,000 unique interactions of different types and holds more than 5,000 human pathway concepts. The database is available through a web server^1^ where queries of genes, proteins, drugs and other types of biomolecules can be made, along with gene and metabolites analysis, such as enrichment and over-representation analysis (Herwig et al., 2016).

ConsensusPathDB holds an integrated network which is comprised of more than 300,000 binary protein–protein interactions (PPIs) representing a comprehensive model of the human interactome. These interactions were scored with a mixture of topology-based and annotation-based measures, such as the ones described in Goldberg and Roth (2003), Kuchaiev et al. (2009), Yu et al. (2010), and Kamburov et al. (2012a). These measures were aggregated into a meta-score using the IntScore (Kamburov et al., 2012b) approach, which combines the individual confidence scores, and provides a final score that better indicates how plausible the interaction is. The PPI network, along with the quality assessment scores, can be downloaded via http://cpdb.molgen.mpg.de/download/ConsensusPathDB_human_PPI.gz.

### ToxDB

ToxDB (Hardt et al., 2016) integrates toxicogenomics data from two large-scale studies, Open TG-GATEs (Uehara et al., 2010) and DrugMatrix (Ganter et al., 2005), with pathway concepts from ConsensusPathDB (Kamburov et al., 2009). It contains a total of 7,464 different treatment data sets, covering 437 drugs, and 2,694 molecular pathway concepts with response scores. Its web interface is available at http://toxdb.molgen.mpg.de/and allows browsing for the effect of a drug treatment on cellular pathway response. The user can also browse for a specific pathway and retrieve the treatments that affect it the most.

## Methods

### Microarray Data Processing

We processed the microarray data sets of the heart tissues that were treated with anthracyclines. The oligonucleotide sequences (oligoprobes) that were downloaded from DrugMatrix were mapped to the rat genome-build and probe sets were redefined using the resource at http://brainarray.mbni.med.umich.edu/CustomCDF such that each probe is assigned to a unique gene, and each gene is associated with a varying number of probes. It has been shown that re-mapping of oligoprobes unambiguously to the latest genome-build increases performance of Affymetrix Gene Chip transcriptomics platforms (Dai et al., 2005). The replicates of the different drug treatments were grouped together, according to their corresponding dosage and time point. The raw data were normalized using the GC Robust Multi-Array method in the R package *gcrma* (Gentry, 2017).

### Orthology Mapping

Rat genes had to be mapped to human genes by orthology, in order to use the human pathway concepts and PPIs from ConsensusPathDB. This was done via the orthology mapping of the Ensembl BioMart repository (Yates and Akanni, 2016). We used only “One2one” and “One2many” homology relationships: if the rat gene has exactly one orthologous human gene, the corresponding rat microarray value is assigned to that human gene. Otherwise, if the rat gene has multiple orthologs in the human genome, the corresponding rat microarray value is assigned to all human paralogs.

### Differential Gene Expression Analysis

The normalized microarray data were analyzed with the R package *limma* (Ritchie et al., 2015) in order to calculate differentially expressed genes (DEGs), i.e., genes that are up- or downregulated significantly when comparing compound treatment against control experiments. It estimates fold-changes and standard errors by fitting a linear model to each gene profile and uses an empirical Bayesian approach to smoothen these errors.

We applied *limma* for every pair of case-control normalized microarray values. Therefore, for every gene, given any drug, dosage and time point combination, we can calculate its fold change value and a corresponding *P*-value. Fold change is computed as the ratio of the mean expression values of treatment and control. *P*-value is the significance of the fold change given the null hypothesis that there is no change in expression between treatment and control.

### Gene Scoring

In order to measure the response of a gene to a drug treatment experiment we use the following scoring scheme:

for every gene *i*, every drug *j* and every time-dosage treatment *k*:

(1) $$\begin{array}{c} S_{\mathrm{ijk}}=\left|\log_{2}r_{\mathrm{ijk}}\right|\left|\log_{10}P_{\mathrm{ijk}}\right| \end{array}$$

Here *r_ijk_* is the fold change between the treatment and the control experiments, and *P_ijk_* is the *P*-value from the differential expression analysis. This score describes a weighted fold change of the gene, such that the more significant the change is, the higher the weight is. Using this scoring scheme allows us taking into consideration the rather low sample size of the experiments, as well as to avoid a pre-selection of the genes based on their *P*-values only. The score serves as a measure of how much the gene was affected by the treatment, regardless of the change in expression (higher or lower expressed in comparison to the control).

### Pathway Scoring

In previous works (Yildirimman et al., 2011; Hardt et al., 2016) we have also defined a pathway scoring scheme, which is based on the scoring of the genes that the pathway is comprised of. Here, we take all available human pathways from ConsensusPathDB, and their associated genes. We compute for each pathway a relative pathway response (RPR) score which serves as a measure for the response of the pathway to the drug, given gene expression data. The higher the RPR score is, the more significant is the response of the pathway to the treatment. A pathway *M_l_* is defined as a set of *m* genes: *M_l_*={*g_1_*,...,*g_m_*}. Given a treatment of drug *j* at a time point and dosage *k*, we can calculate the pathway score:

(2) $$\begin{array}{c} M_{l,j,k}=\frac{1}{m}\sum_{g_{i}\in M}S_{\mathrm{ijk}} \end{array}$$

Where *S_ijk_* is the gene score of gene *i*, as defin drug *j* and the time-dosage *k* is calculated by dividing the pathway score by ${\bar{M}}_{j,k}$ the median of all pathway scores, given drug *j* and time-dosage *k*:

(3) $$\begin{array}{c} RPR_{l,j,k}=\log_{2}\left(\frac{{\bar{M}}_{l,j,k}}{{\bar{M}}_{j,k}}\right) \end{array}$$

In addition, we computed RPR scores for all pathways in all the different experimental conditions and derived a background distribution. This background distribution is used to judge the significance of a given RPR score and reflects the response of the pathway to the experimental condition.

### Network Module Analysis

A network module analysis was carried out by applying the HotNet2 (Leiserson et al., 2015) algorithm, which was originally developed to identify significantly mutated subnetworks in cancer in PPI networks based on somatic mutations data. The algorithm takes as input a score vector *S* =(*S_1_*,...,*S_n_*), where *n* is the number of genes, and a graph *G*=(*V, E*). The gene scores are computed context dependent (see below), and the graph represents a PPI network, where each node corresponds to a protein coding gene, and each edge to an interaction between their respective proteins. HotNet2 then applies an insulated heat diffusion process that includes the following steps:

1. Heat diffusion – at each time step heat is diffused from every node *i* to every one of its neighbors *j*. The amount of heat that will be placed on node *j* given the initial heat on node *i* is given by the entry (*i,j*) of the diffusion matrix *F*, which is defined by:

(4) $$\begin{array}{c} F=\beta {\left(I-\left(1-\beta \right)W\right)}^{-1} \end{array}$$

$W_{i,j}=\frac{1}{deg\left(j\right)}$ if *(i,j)* are neighbors, otherwise 0.

The parameter *β* is an insulating parameter and *W* is the normalized adjacency matrix of the input graph *G* such that de*g*(*j*) is the degree (number of neighbors) of node *j*. I is the identity matrix.

2. Exchanged heat - the amount of heat that diffuses from node *j* to node *i* when heat *S_j_* is placed on node *j* is given by the exchanged heat matrix *E* which is defined by:

(5) $$\begin{array}{c} E=FD_{s} \end{array}$$

Where *D_s_* is a diagonal matrix with the entries of *S*.

3. Identification of subnetworks - a new weighted directed graph *H* is created using the nodes *V*. Node *i* will be connected to node *j* in this graph if *E*(*i,j*) > δ, where δ is a minimum edge weight parameter, and their respective edge will have a weight equal to *E*(*i,j*). Then, the strongly connected components of *H* are identified and are selected to be the final subnetworks.

4. Statistical test for the subnetworks – a two-stage statistical test, that is described in the original HotNet algorithm (Vandin et al., 2011, 2012), is applied to determine the significance of the number and the sizes of the subnetworks.

To identify functional modules that are associated with the different drug treatments we used the HotNet2 algorithm (Leiserson et al., 2015) that is available at http://compbio.cs.brown.edu/projects/hotnet2/. Since the first step of the algorithm depends only on the graph G and the chosen parameter *β*, we calculated the diffusion matrix F for the high-confidence ConsensusPathDB PPI network, while choosing *β* = 0.5. For the scoring of the genes, we used our own data-derived scores: for each drug and treatment, we used as input their gene scores, as described in Equation 1. The output of the HotNet2 algorithm depends largely on δ, the minimum edge weight parameter. The lower its value, the larger are the subnetworks. HotNet2 outputs four different results, for four different δ values, which are chosen based on a permutation test in their algorithm [for further details see (Leiserson et al., 2015)]. In this work we chose for further analysis the subnetworks which are resulted when taking the smallest δ parameter from the output of the HotNet2 algorithm.

### Over-Representation Analysis (ORA)

ConsensusPathDB allows performing over-representation analysis (ORA) with different functionally relevant gene sets (Herwig et al., 2016). Given a set of genes, proteins or metabolites over-represented sets are searched among three pre-defined categories: (1) network neighborhood-based sets, (2) pathway-based sets, and (3) Gene Ontology (GO)-based sets. According to the hypergeometric test, a *P*-value is calculated based on the number of identifiers that are present in the given set and in the pre-defined sets. As background, the user can choose another set of identifiers, for example all genes that were measured in the experiment, or simply use all entities that are annotated in ConsensusPathDB. In our work, ORA was used to identify only the pathway based enriched sets, choosing all possible pathways from ConsensusPathDB and applying a *P*-value cutoff of 0.01. As background, we used the full list of genes that were measured in the corresponding experiment.

## Results

### Workflow for Analyzing Toxicogenomics Data in the Context of Networks and Pathways

**FIGURE 1:**
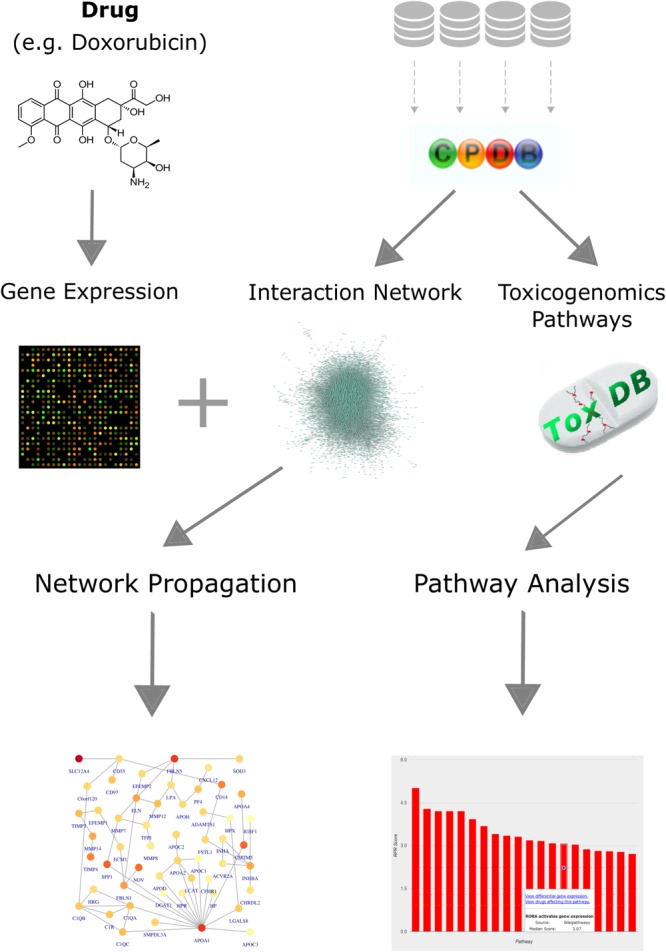
Workflow for analyzing toxicogenomics data at the network and pathway level. Gene expression data, following a drug perturbation, is collected and analyzed to identify differentially expressed genes. Pathway concepts and interaction network are extracted from ConsensusPathDB. Identification of relevant pathways is done via ToxDB, while functional modules are detected by applying a network propagation algorithm that combines both the gene expression data and the PPI network. The workflow allows us to identify: (i) differentially expressed genes that could be candidates for further experiments, (ii) relevant pathways that are disrupted in response to the treatment, and (iii) network toxicity modules that hold functional information about the mechanisms of action.

We established a computational workflow for analyzing toxicogenomics data by incorporating pathway and network information using different complementary approaches in order to gain functional information from gene expression data (Figure 1). We exemplify the results on the four anthracyclines drugs: daunorubicin (DAU), doxorubicin (DOX), idarubicin (IDA), 4-epidoxorubicin (EPI). We also applied our analysis to three other anti-cancer drugs that are known to cause cardiotoxicity: cyclophosphamide (CYC), ifosfamide (IFO), and imatinib (IMA). We compare our results for the anthracyclines with our results for these drugs in order to identify differences and commonalities and distinguish the effects that are explicit to anthracyclines. The workflow is based on the results of a differential expression analysis, and combines pathway and network information from both ConsensusPathDB and ToxDB. It begins with an over-representation analysis for the DEGs, using pathway concepts that are collected in ConsensusPathDB, in order to assign a biological function to the most significantly changed genes. Next, it continues with a pathway analysis using ToxDB, extrapolating from DEGs to the entire gene expression response and from gene lists to pathway concepts. Using molecular interaction information from ConsensusPathDB, the workflow also includes a PPI network construction and an analysis that applies a network propagation algorithm which combines the DEGs with the PPI network. Finally, it is able to identify subnetworks that we define as drug toxicity modules.

### Assigning Biological Function to Gene Lists With Over-Representation Analysis

**FIGURE 2:**
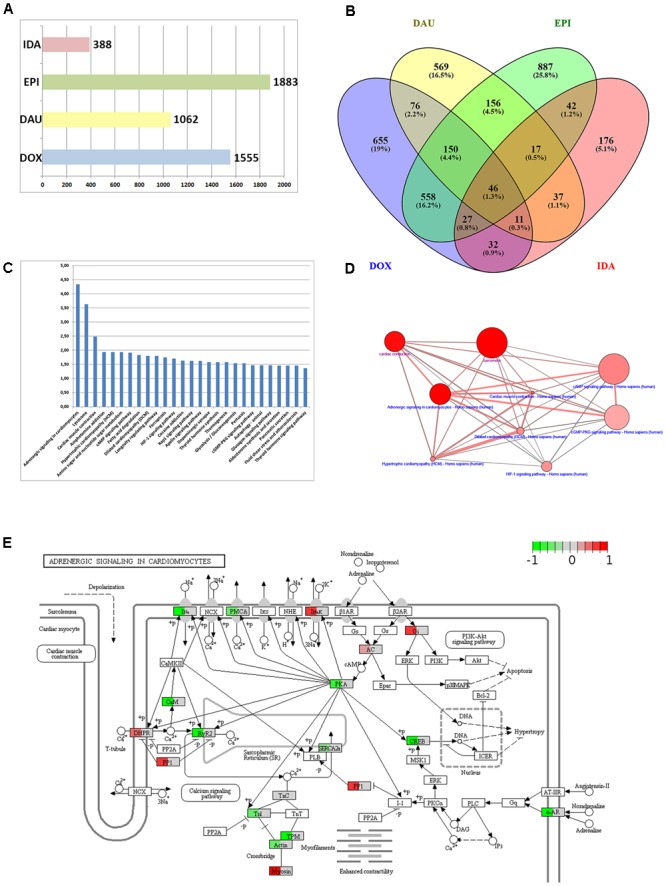
Anthracycline over-representation analysis. **(A)** Summary of the number of DEGs from the different experimental conditions: DOX (3 mg/kg at 1, 3, and 5 days), DAU (3.25 and 2 mg/kg at 1, 3, and 5 days), EPI (2.7 mg/kg at 1, 3, and 5 days), and IDA (0.625 mg/kg at 1, 3, and 5 days). **(B)** VENN diagram of DEGs with respect to the four compound treatments. **(C)** 27 KEGG pathways that were found significantly over-represented with respect to the 1555 DEGs after DOX treatment using the Fisher test statistic with the ConsensusPathDB. *Y*-axis = –log10(*P*-value). **(D)** Interdependency of significant pathways from **(C)** (blue label) and GO categories (magenta label) computed with ConsensusPathDB. Size of balls indicates pathway size, shade of balls indicate overlap with DEG list. **(E)** “Adrenergic signaling in cardiomyocytes” pathway found significantly over-represented (*P* = 3.49E-07) and expression data of 32 DEGs overlaid with the pathway. Mapping of gene expression fold-changes to pathway has been done with Pathview (Luo et al., 2017).

A first step in functional interpretation of toxicogenomics results is to interrogate the lists of DEGs (see Methods) for known annotation sets such as pathways or GO terms using Fisher’s test or similar statistics (see Methods). Summarizing the different experiments (time points and dosages) for the four anthracyclines (DOX, DAU, EPI and IDA) results in 1,883 DEGs for EPI, 1,555 for DOX and 1,062 for DAU whereas IDA shows a much weaker response with 388 genes (Figure 2A). In all cases, human genes were inferred based on homology mapping of the corresponding rat microarray probes. All anthracyclines were administered at maximum tolerated doses (MTDs) and, thus should be of comparable toxicity (DOX 3 mg/kg; DAU 3.25 mg/kg; EPI 2.7 mg/kg and IDA 0.625 mg/kg). Also, it has been shown that gene expression signatures are predictive of toxicity and that number of DEGs is indicative of phenotypically observed injury of the organ (Paules, 2003; Andersen et al., 2008; Holmgren et al., 2015) which has given rise to the concept of phenotypic anchoring, i.e., the association of gene expression signatures to toxic phenotypes. The difference in DEGs between DOX and IDA is in line with previous findings: For example, Platel et al. (1999) showed that in rat the MTDs for DOX and IDA were 3 mg/kg and 0.75 mg/kg, i.e., comparable to the levels used in the DrugMatrix screen, and that at these MTDs IDA showed significantly lower cardiotoxicity than DOX. Anthracyclines show highly specific response at the gene expression level (Figure 2B) with 40–50% of all DEGs specific for a certain compound. The strongest relative agreement in gene expression response was observed between DOX and EPI (45%), whereas the relative agreement between IDA and the other three compounds is much lower (12–15%). DOX response can be characterized at the pathway level using ORA analysis (see Methods). Figure 2C exemplifies the results for DOX using gene sets that represent KEGG (Kanehisa et al., 2012) pathways and that might reflect the cell’s response to the drug treatment. In total, 27 KEGG pathways are significantly over-represented (Q-value < 0.05 and at least 10 DEGs overlapping with the pathway gene set). A number of disease gene sets have been identified such as “Hypertrophic cardiomyopathy” (Q = 0.0115), “Dilated cardiomyopathy“(Q = 0.0158) or “Cardiac muscle contraction” (Q = 0.0032). Interestingly, these pathways were also found in a recent study investigating cardio-toxicity in human pluripotent stem cell derived-cardiomyocytes (Maillet et al., 2016) and thus seem to extrapolate from rat *in vivo* to human *in vitro* studies. The top-enriched pathway in our setting is “Adrenergic signaling in cardiomyocytes” (*Q* = 4.54E-05). 32 genes of that pathway are differentially expressed including troponins (*TNNC1* and *TNNI3*), tropomyosins (*TPM1* and *TPM2*), and other well-known toxicity-associated genes such as *RYR2* (ryanodine receptor 2). Figure 2D displays the interdependencies of these and other disease-related gene sets.

An important feature in the analysis is the visualization of the gene expression changes in the pathway map. Pathway maps can be retrieved by pathway resources such as KEGG. There are many tools that allow visualizing gene expression fold-changes on these pathways which is exemplified in Figure 2E with the “Adrenergic signaling in cardiomyocytes” and the expression fold changes of the DOX treatment.

### From Genes to Pathways – Pathway Analysis Using the ToxDB

**FIGURE 3:**
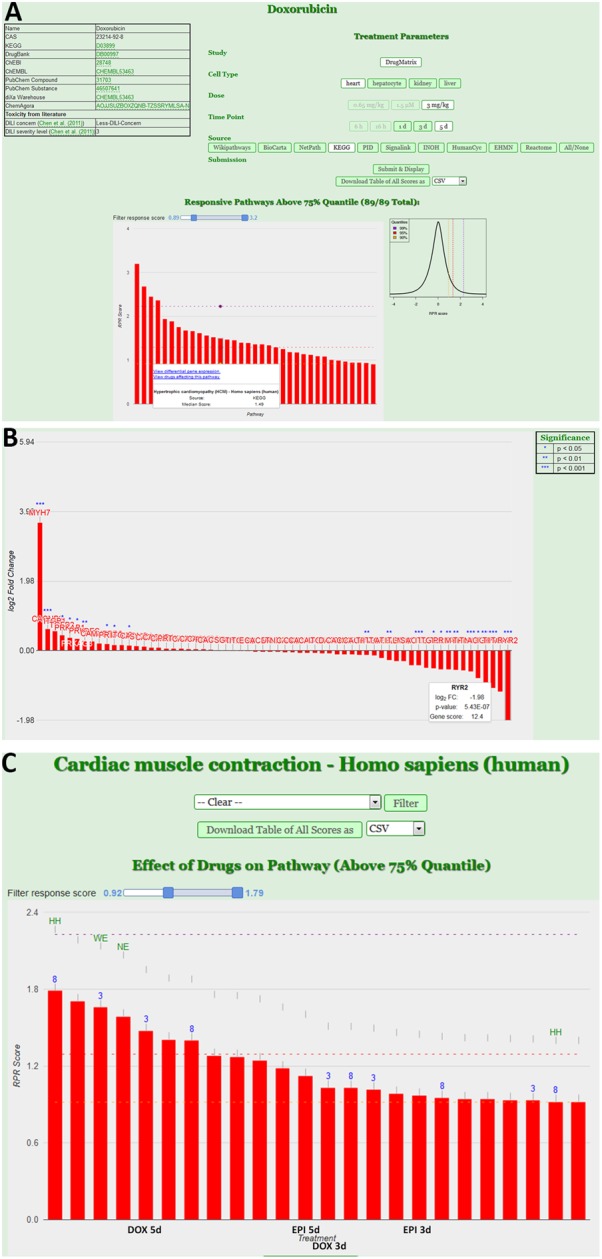
Pathway analysis using the ToxDB. **(A)** Drug view contains links to chemical information (top left), specification of the experimental data (top right) and the display of the response pathways for that experiment in form of an interactive bar plot. Bars indicate the strength of the response, with mouse over the user can display further information of the pathway. The background distribution of the response scores is displayed as density plot next to the plot. **(B)** Gene view. Once a pathway is selected, user can inspect the experimental results of the genes. In this case the log2 fold-changes of the genes associated with the pathway “Hypertrophic cardiomyopathy (HCM)” from the KEGG database are shown. Stars indicate differentially expressed genes (DEGs). **(C)** Pathway view. Users can infer specific pathways (here “Cardiac muscle contraction”). The interactive bar plots represents the response of that pathway in different experiments (e.g., DOX and EPI at 3 and 5 days experiments).

The next level of analysis is to extrapolate the gene expression values from single genes to entire pathways. We have built a tool, ToxDB that combines gene expression data and pathway concepts. ToxDB builds on three components: (i) a comprehensive collection of pathway concepts along with drug treatment microarray data, (ii) a numerical method to compute pathway responses from genome-scale expression data, and (iii) a web interface that allows user interaction. By this procedure each pathway is assigned a numerical value that reflects its response to the treatment (see Methods). ToxDB contains pre-calculated pathway scores for ca. 2,700 different pathways and ca. 7,500 experimental conditions mainly extracted from two large toxicogenomics studies, TG-GATES and DrugMatrix (see Methods). A background distribution of pathway scores is used to infer statistical significance. ToxDB can be used in different views. The drug view allows drug centric analysis: by selecting a compound, for example DOX, and a specific experiment all responding pathways can be viewed (Figure 3A). By further clicking on a specific response pathway [here “Hypertrophic cardiomyopathy (HCM)”] the expression results of all genes can be inspected that are associated with this pathway (Figure 3B). DEGs of this pathway are known cardiac-relevant genes such as *MYH7* (myosin, heavy chain 7, cardiac muscle, beta; log2-FC = 3.65, *P* = 9.05E-06), *DES* (desmin; log2-FC = 0.264, P = 7.55E-02), *TPM4* (tropomyosin 4; log2-FC = -1.07, P = 9.14E-04), or *RYR2* (ryanodine receptor 2; log2-FC = -1.98, *P* = 5.43E-07). A second view is the pathway view: the user can select a single pathway (here “Cardiac muscle contraction”) and as a result all experiments are shown in which this pathway responded significantly (Figure 3C). Pathways can be selected from ten different resources which comprise most widely used pathway resources such as KEGG, Reactome or BioCarta. It can be seen from the view that anthracycline experiments (DOX and EPI at different time points) are among the compounds that induce the most significant responses of cardiac muscle contraction.

### Protein–Protein Interaction Network Construction

Protein–protein interaction networks are typically used as scaffolds for drawing network propagation of gene expression data on. The underlying argument is “guilt-by-association,” i.e., the assumption that genes/proteins that interact with each other usually share function and, thus, that network modules computed from these PPI networks amplify functional information. Thus, the PPI network needs to be properly selected in the sense that it should be sufficiently comprehensive and that the false-positive rate of interactions should be low.

**FIGURE 4:**
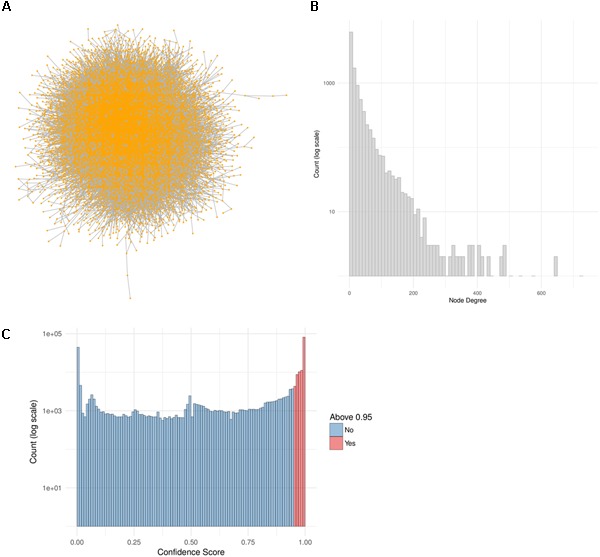
PPI Network construction from ConsensusPathDB. **(A)** High-confidence PPI network extracted from the ConsensusPathDB database with 10,707 nodes and 114,516 undirected edges. **(B)** Node degree distribution of the PPI network. **(C)** Distribution of all IntScore confidence scores from all ConsensusPathDB unique interactions. In blue are the confidence scores below 0.95 and in red are those above it. The high-confidence network includes only the red interactions.

In this work we make use of the PPI network from ConsensusPathDB (release 32) and reduce it to a high-confidence network by taking only the interactions with a confidence score of 0.95 or higher (Figure 4C). This network is comprised of 10,707 proteins and 114,516 unique interactions (Figure 4A). Biological networks are normally characterized with a power law distribution of the node degree (Barabasi and Oltvai, 2004). This means that most of the nodes in the network are only connected to a few other nodes, while a small majority is very highly connected, with more than 400 neighbors (Figure 4B). We make use of this high-confidence interaction network in our workflow in order to identify subnetworks that are highly relevant to the drug treatments.

### Toxicity Network Modules Are Identified by Applying Network Propagation

**FIGURE 5:**
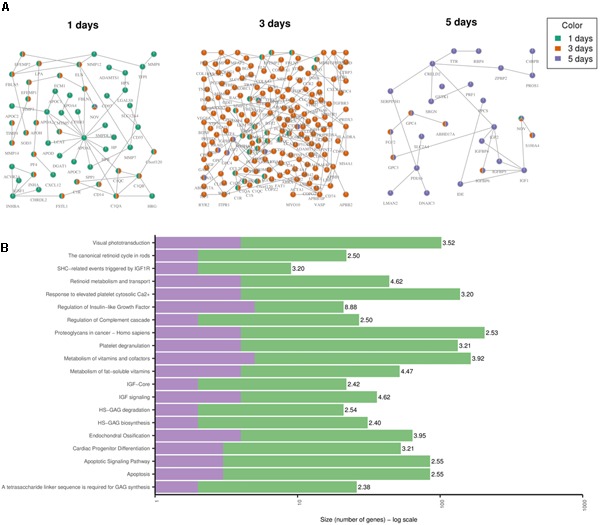
Toxicity Modules. **(A)** Toxicity modules were identified using the HotNet2 propagation algorithm for DOX drug treatments after 1, 3, and 5 days. Each node corresponds to a protein coding gene (the nodes are named using their HGNC symbol) and each edge is an interaction as defined by the PPI network of ConsensusPathDB (see Methods). The colors of the nodes indicate the time point. Nodes that are colored in green only are present in the “1 day” module only. In the same way for orange and purple. Nodes that are colored in two colors are present in the two corresponding modules. Nodes that are colored in three colors are present in all modules. **(B)** The top 20 over-represented pathways for the “5 days” module, based on the ORA of the genes in the module with ConsensusPathDB (see Methods). The purple color represents the overlap of genes from the pathway and the module. The green are the rest of the genes from the pathway (that are not in the module). The number next to each bar displays the significance of the over-representation [–log10(*Q*-value) of the corresponding pathway].

Toxicity modules were calculated using the HotNet2 algorithm for each drug and time point independently. A detailed list of all the toxicity modules is provided in Supplementary Table 1. Here we discuss the results when using the gene expression values for DOX only. The modules for the other anthracyclines are provided in Supplementary Figures 1–3. Since the drug treatments of DOX were measured three times over the course of 5 days, we derived one module for each one of the time points (Figure 5A). Looking at each one of the modules, and at all of them together, allows us to analyze the changes over time. We identified that the effect becomes stronger after 3 days, as the size of the module grows, but also that it is again much lower after 5 days. This could be due to the toxic effect of the drugs on the cells, i.e., the cells might already be dying. We confirmed this by looking at the over-represented pathways for the genes in the “5 days” module (Figure 5B). We observed two pathways that indicate cell death: “Apoptosis” and “Apoptotic Signaling Pathway.” In addition, we identified another pathway that might be involved in cardiotoxicity: “Cardiac Progenitor Differentiation” (from the WikiPathways database). This pathway includes several factors that are involved in cardiac differentiation, such as *TNNI3* that we also detected as differentially expressed, and is based on two recent reviews (Burridge et al., 2012; Stillitano et al., 2012). The module also includes the genes *IGF1* and *IGF2* that are involved in the differentiation of immature cardiomyocytes and have been associated with cardiac hypertrophy (Wang et al., 2012). Other genes that might be involved in cardiotoxicity and are present in the “1 day” and “3 days” modules are *APOA1*, that have been previously associated with hereditary amyloid cardiomyopathy (Hamidi Asl et al., 1999), and *ELN* that has been involved in both progressive aortic valve malformation and latent valve disease in mice (Hinton et al., 2010).

### Network Modules Amplify Functional Information

**FIGURE 6:**
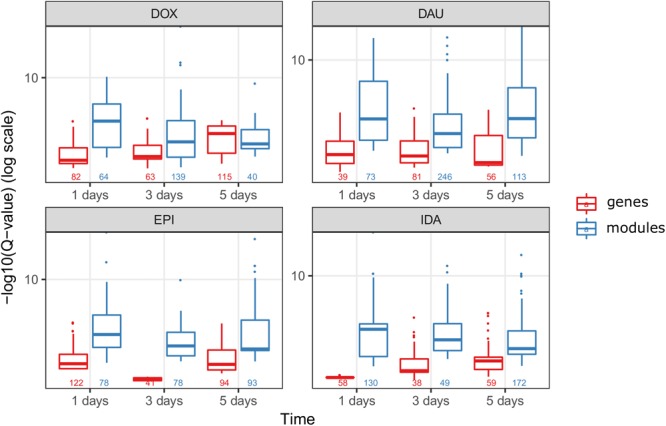
Network modules amplify functional information. We compared the scores of the over-represented pathways when using the highly scoring genes (score >99th quantile) (in red) and when using the genes from the HotNet2 modules (in blue), for all four anthracyclines and in the three time points of the experiments. The scores of the pathways are the –log10(*Q*-value) of the *Q*-values from the ORA that was done via ConsensusPathDB (*Q*-values are the FDR corrected *P*-values from the hypergeometric test). Below the boxplots, the numbers indicate the number of the significantly (*P*-value < 0.01) over-represented pathways, for each one of the conditions.

We compared the over-represented pathways when using only the high scoring genes (genes with a score above the 99th quantile of the background distribution of all scores), and when using the genes from the network modules (Figure 6). In 8 out of 12 drug-treatment conditions the enrichment scores when using the genes from the network modules, were higher than the scores when using the high scoring genes only. Furthermore, when comparing the significance of the enrichment, by looking at the means of the Q-values (FDR corrected *P*-values), in all but one case we observed a higher enrichment when using the genes from the modules. This suggests that the network modules are enriched in more functional information, and therefore they serve as a powerful mean for studying systemic processes, such as drug induced toxicity.

### Differences and Commonalities Between Anthracyclines and Other Chemotherapeutic Drugs

**FIGURE 7:**
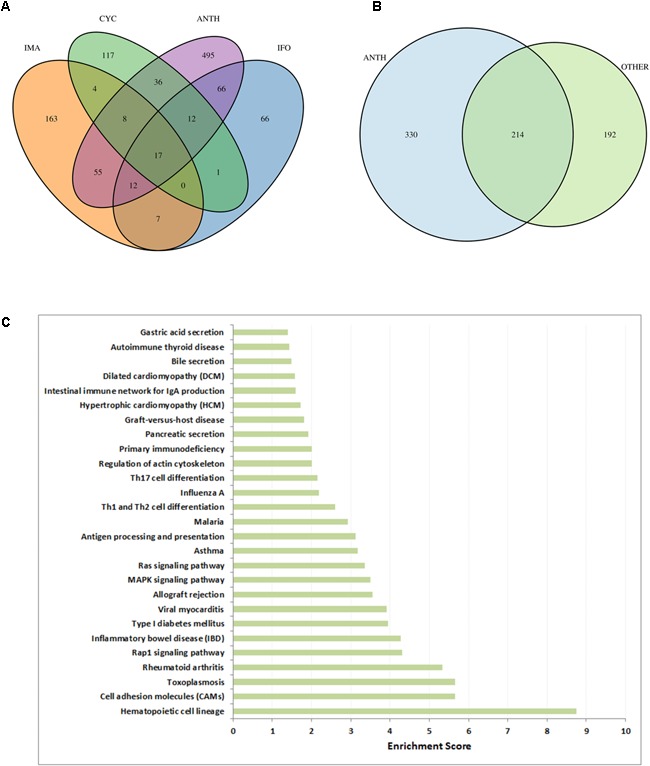
Cardiotoxic effects of anthracyclines in comparison to other drugs. **(A)** VENN diagram of high-scoring genes (genes with a score above the 99th percentile) with respect to the three other compounds (CYC, IFO, and IMA) and all anthracyclines together (ANTH). **(B)** VENN diagram of genes in the toxicity modules, with respect to all anthracyclines (ANTH) together and all other drugs together (OTHER). **(C)** Significantly enriched KEGG pathways (*P* < 0.01) with the 330 genes that are contained in the computed anthracycline toxicity modules and not contained in the toxicity modules of the other cardiotoxic drugs. Bars indicate an enrichment score computed as –log10(*Q*-value), where *Q*-value is the FRD-corrected *P*-value of the enrichment.

To assess the specificity of our results for anthracycline-induced cardiotoxicity, we applied the same workflow (Figure 1) to three other anti-cancer drugs which are known to cause cardiotoxic phenotypes: cyclophosphamide, ifosfamide and imatinib (see Materials). Looking only at the high scoring genes (genes with a score above the 99^th^ quantile), with gene scores computed according to section 3.4, we observe hardly any common genes between the three drugs (Figure 7A). Interestingly the 17 genes that are common between these three drugs are also common with all the other anthracyclines drugs. However, when we compare the genes that are present in the toxicity modules of these drugs and the toxicity modules of all anthracyclines (Figure 7B) we detect 214 common genes. This highlights the fact that the network propagation approach amplifies gene expression responses toward relevant cardiotoxic mechanisms and phenotypes that are shared by the different drugs so that different gene expression responses can result in similar pathway responses. Evidently the number of genes in the anthracyclines modules is much higher as they are derived from more drugs and experiments, but nonetheless the percentage of number of genes that are shared is much higher. This could again indicate to the functional information that is inherent within the toxicity modules, which might suggest to the mechanisms that are involved in causing the toxic effect.

In order to identify biological functions that are specific for anthracyclines we performed enrichment analysis with the set of 330 genes that are solely part of the anthracycline toxicity modules (Figure 7C). We observe enrichment of cardiac disease pathways such as “Viral myocarditis,” “Hypertrophic cardiomyopathy,” and “Dilated cardiomyopathy,” mainly through the inclusion of ITGB and TGFB gene family members and RYR2. Another strong signal is the presence of immune response pathways. It is well-known that anthracycline treatment can induce systemic inflammation mediated through interleukins (Mills et al., 2008; Sauter et al., 2011). Interestingly, many inflammatory and immune response pathways are enriched with the anthracycline toxicity modules, in particular through interleukins (IL1A, IL12A, IL12B, IL23A, IL33, and IL27RA) that are not included in the modules of the other drugs.

## Discussion

Combining the information from ConsensusPathDB and ToxDB, including pathway concepts and a PPI network, together with experimental data, allows for a more comprehensive view of the effects of the drug treatments. Firstly, by using ToxDB we are able to identify pathway concepts and by that suggest specific mechanisms that may be either the cause or the consequences of the toxic effects. In addition, by using the information from the PPI network and a propagation algorithm, we can also identify specific interactions that could be highly relevant for further experiments. These network modules carry out more functional information, since their genes and interactions represent parts of different pathways, and thus they are enriched in more information about specific biological mechanisms. Indeed, by propagating perturbation data across a network it is possible to gain information not only for the genes that were actually measured by the experiment but in addition also for the genes that haven’t been measured experimentally but that are connected with many measured neighbors in the network.

When looking only at DEGs, it is very difficult to describe the toxic effects of a drug given a specific treatment. Usually, this list of genes is comprised of hundreds of possible candidates, and it can be very challenging to distinguish which ones are involved in causing toxicity. Other works have tried to reduce the number of genes by looking at a smaller toxicogenomics space (Kohonen and Parkkinen, 2017). By defining a more complex gene score, we were able to reduce the number of genes such that it becomes easier to extract plausible candidates for further studies. Furthermore, by applying a network propagation scheme to the gene scores and the high-confidence PPI network, we were able to both reduce the list even further, and also identify functional modules within PPI networks. These functional modules can better reflect the mechanisms that lead to toxicity, as they contain not only the obvious candidate genes based on the differential expression analysis, but also other genes that might be associated with the toxic effect, and are also connected to the more significantly changed genes.

ConsensusPathDB is a meta-database that agglomerates information from multiple resources and therefore includes different kinds of interactions: protein–protein, genetic, metabolic, signaling, gene regulatory and drug-target. In addition, it also holds information about biochemical molecules and pathways. The high confidence PPI network that we have constructed is comprised solely of highly scored protein–protein interactions that are extracted from several resources, such as BIND, INTACT, HPRD. However, the ORA that is provided within ConsensusPathDB, searches for over representation of genes within cellular pathways. These pathways are derived from other resources, such as KEGG, Reactome, WikiPathways, etc. The different resources are completely independent data sets and the ConsensusPathDB simply serves as a common analysis platform. Therefore, when we apply the ORA to the extracted network modules, we can identify how enriched they are not only with protein–protein interactions, but also with pathway information. As we have illustrated in Figure 6, network modules contain not only protein–protein interaction information (that is inherent within its structure) but also are enriched in other functional information that is represented in various pathways.

It should be noted that besides the described publicly available tools for pathway annotation and analysis, there are commercially available tools that hold functionality for pathway and network analysis such as IPA (Ingenuity/Qiagen), TransPath (geneXplain), or MetaCore (Thomson Reuters). These and other commercial and publicly available tools can be used to construct suitable molecular networks and perform enrichment analysis and module computation. A survey of databases and resources is given by Pathguide (Bader et al., 2006), a recent review and comparison of pathway tools has been published for example for metabolomics data (Marco-Ramell et al., 2018).

Toxicology studies often explore the effects of compounds over time and varying dosages (Hartung, 2009). Here, we analyzed gene expression levels for three different time points: after 1, 3, and 5 days. Every time point experiment was independently compared to the control experiment, such that a network module was constructed for every time point. To discern the effect over time, we compared between the modules, and determined the possible changes due to time. We were able to identify the toxic effect over time by looking at the different modules and also the genes within the module that could implicate the pathways that are leading to toxicity. In the future, one could try to first integrate the experimental data from the different time points, such that the change in expression levels over time is taken under consideration. For example by applying a mathematical model to detect differential expression over time, like the one suggested by Conesa et al. (2006). We could further use the results of such model and incorporate them into the network propagation algorithm in order to identify a module that encompasses data from all the time points together.

The approach we applied in this work consists of three main components: gene expression analysis, a PPI network and a network propagation algorithm. All of these have several alternatives, and could be further incorporated in future analysis. Firstly, the PPI network can be replaced with other genetic interaction networks, for example a gene regulatory network that is derived from experimental data (Zheng and Huang, 2018). Secondly, different types of experimental data can be used for ranking the genes and using their ranks as scores for the chosen propagation algorithm. Gene expression values from RNA-seq experiments could easily be investigated in the same manner, along with protein abundance data, mutation data or epigenetic data. Finally, there already exist different approaches for applying propagation algorithms to detect network modules. Here we have chosen to use the HotNet2 algorithm, but several others, like the ones in the review by Cowen et al. (2017), might also be considered.

In our work we focus on anthracyclines, a group of commonly used chemotherapy drugs. We used the data that are available in the DrugMatrix (Ganter et al., 2005) database and applied our workflow (Figure 1). This workflow could easily be applied to other data resources as well as other groups of drugs. Some previous works have already been developed to analyze toxicogenomics data from DrugMatrix and were applied for identifying different types of drug induced toxicities. For example, Tawa et al. (2014) characterized liver induced drug toxicity by identifying gene co-expression modules that are associated with a toxic response. They defined these gene modules using six different methods, including Pearson correlations and PPI information. A similar approach was also applied to identify gene co-expression modules for kidney induced drug toxicity (AbdulHameed et al., 2016). In another work, AbdulHameed et al. (2014) also tried to identify liver induced drug toxicity by integrating toxicogenomics data with pathway and PPI network information. They performed a differential expression analysis and identified relevant gene modules by applying the KeyPathwayMiner (Alcaraz et al., 2012) algorithm. Other network based approaches have also been suggested for the analysis of toxicogenomics data from the DrugMatrix database. For instance, Sutherland et al. (2017) have constructed gene co-expression networks using WGCNA (Zhang and Horvath, 2005) and associated modules with different drug toxicity phenotypes. Mulas et al. (2017) compiled a pipeline for network comparison and used it to identify drugs with similar toxicity profiles. In our workflow, we chose to apply a network propagation algorithm that is based on a random walk model. We showed that this approach allows for the identification of drug toxicity modules that are highly enriched in functional information and provide new insights into the toxic causing mechanisms.

Gene expression signatures have been associated with toxicity phenotypes with the concept of phenotypic anchoring (Paules, 2003). Here, the idea is that specific signatures emerge over time and dose that can be related to distinguishable phenotypes. We have observed that, for example the number of DEGs in DOX and IDA at MTDs reflect previously observed differences in the toxicity of both compounds. Additionally, when comparing enrichment scores in heart-related diseases pathways, DOX appears as the most toxic compound followed by EPI, while IDA and DAU show basically no enrichment in these pathways (Supplementary Figure 4).

Associating genotype with phenotype, and specifically predicting a toxic phenotype that rises due to drug treatment, still remains an intricate challenge. Integrating experimental data with prior knowledge in the form of biological networks, as suggested in our work, is a suitable step when trying to describe the molecular effects of drug treatments. However, there is still much to be improved. The PPI networks still hold a high bias in interactions due to annotation (Schramm et al., 2013; Luecken et al., 2018) and will keep getting refined as our understanding of the biological systems increases. Better experimental techniques become more and more available, and data from those will need to be integrated for an even more comprehensive analysis (Hasin et al., 2017; Yan et al., 2017; Karczewski and Snyder, 2018). And finally, better computational approaches for differentiating between cases and controls, as well as for analyzing big networks such as PPIs, are still to be developed.

## Author Contributions

GB developed the workflow, performed parts of the data analysis, and wrote the manuscript. RH conceived the study, performed parts of the data analysis, and wrote the manuscript.

## Conflict of Interest Statement

The authors declare that the research was conducted in the absence of any commercial or financial relationships that could be construed as a potential conflict of interest.

## Footnotes

1 http://consensuspathdb.org
