# Supplementary figures and images for: Network and Pathway Analysis of Toxicogenomics Data
Source: Front Genet. 2018 Oct 22;9:484. doi: 10.3389/fgene.2018.00484 (PMC6204403; doi:10.3389/fgene.2018.00484)

## DAU

## 1 days

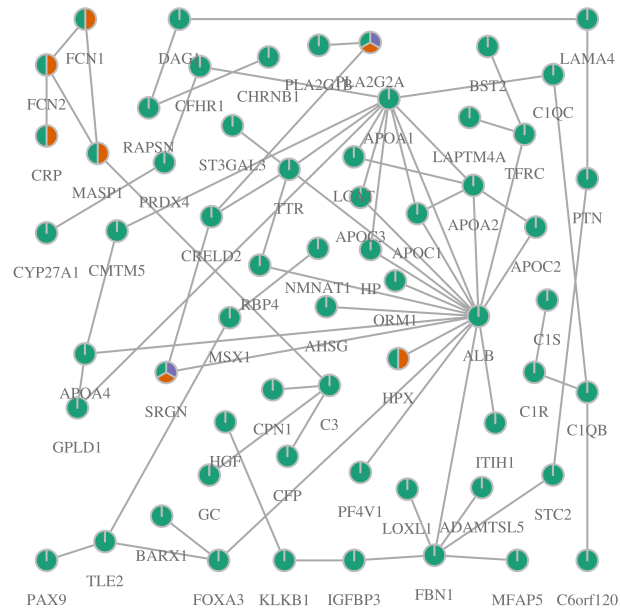

## 3 days

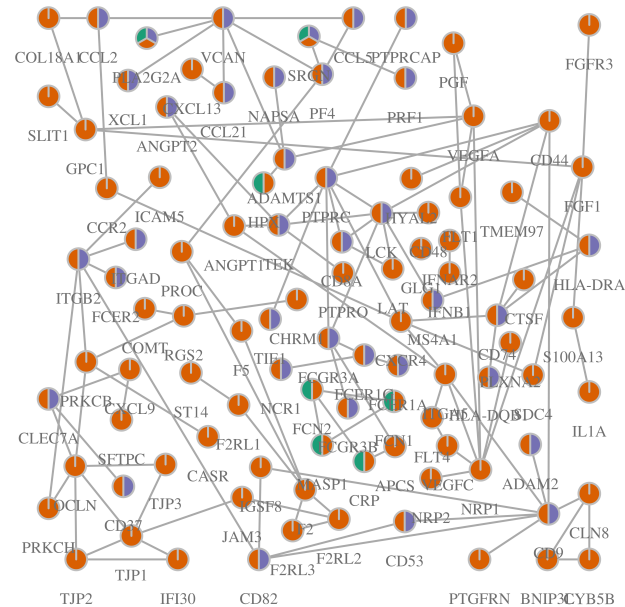

## 5 days

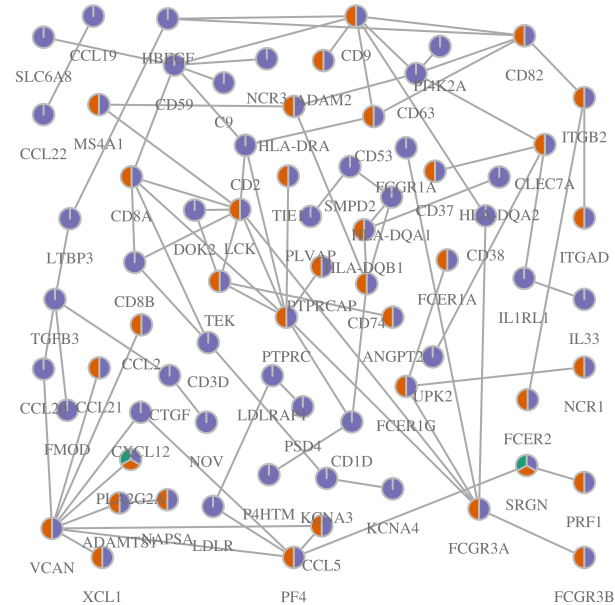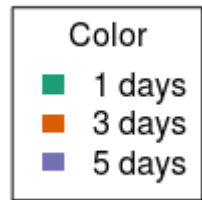

**Supplementary Figure 1:** Toxicity modules for DAU drug treatments

Supplement: Supplementary file 2 [file Image_1.pdf]

# EPI

1 days

3 days

5 days

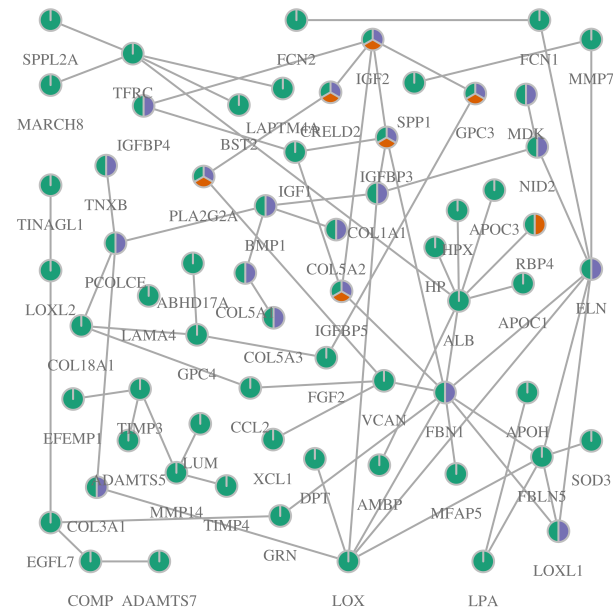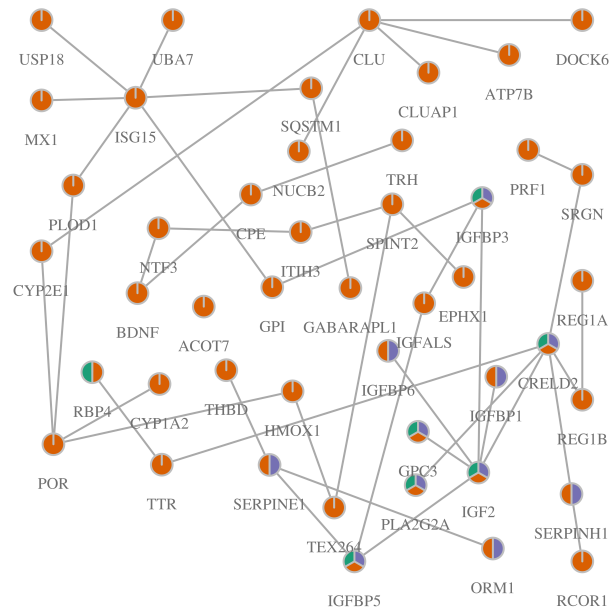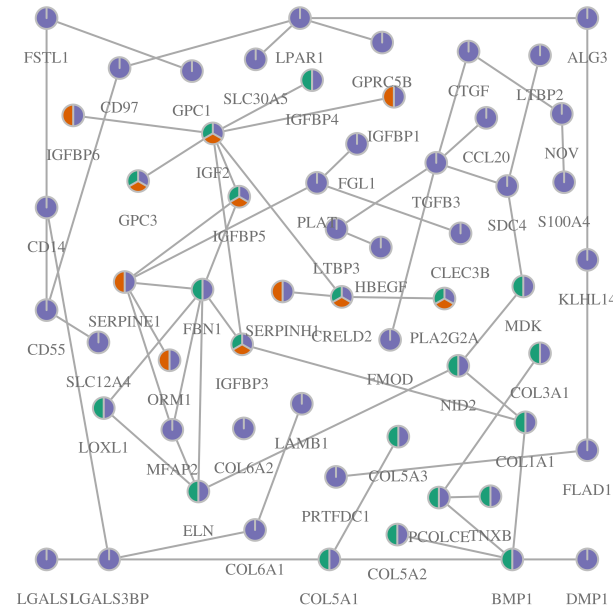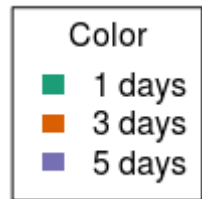

**Supplementary Figure 2:** Toxicity modules for EPI drug treatments

Supplement: Supplementary file 3 [file Image_2.pdf]

# IDA

1 days

3 days

5 days

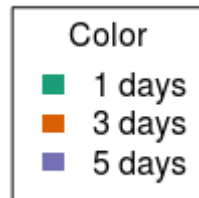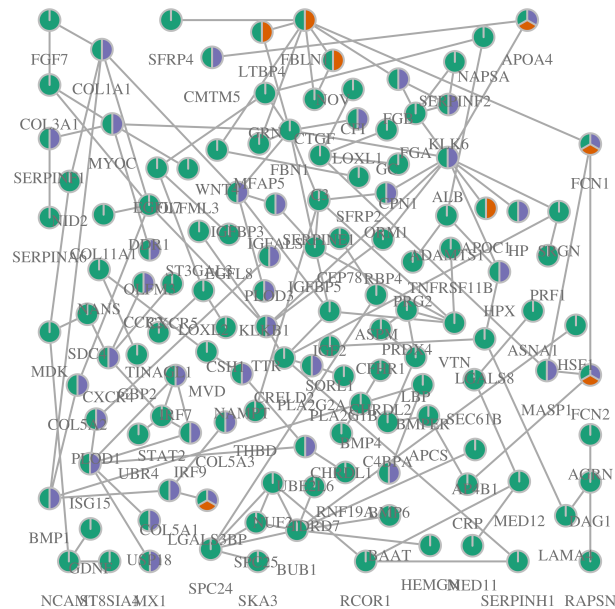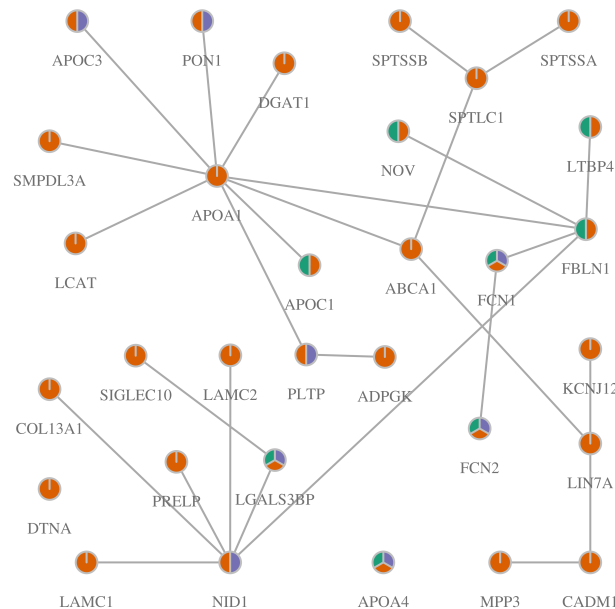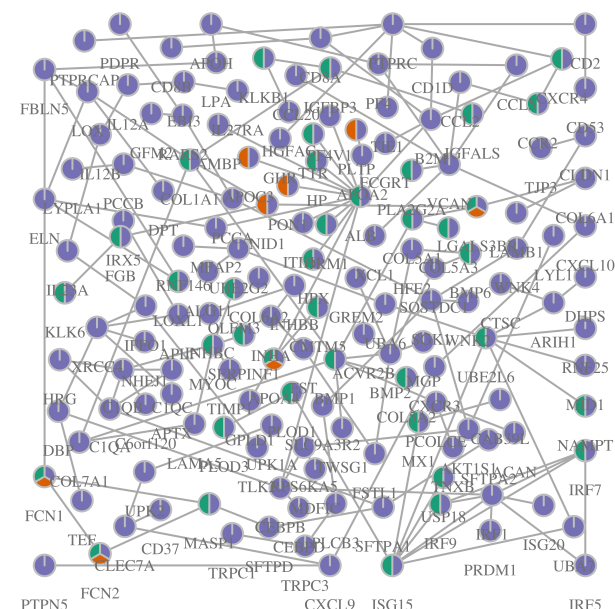

**Supplementary Figure 3: Toxicity modules for IDA drug treatments**

Supplement: Supplementary file 4 [file Image_3.pdf]
