# Supplementary material for: Network and Pathway Analysis of Toxicogenomics Data
Source: Front Genet. 2018 Oct 22;9:484. doi: 10.3389/fgene.2018.00484 (PMC6204403; doi:10.3389/fgene.2018.00484)
Supplement: Supplementary file 5 [file Image_4.pdf]

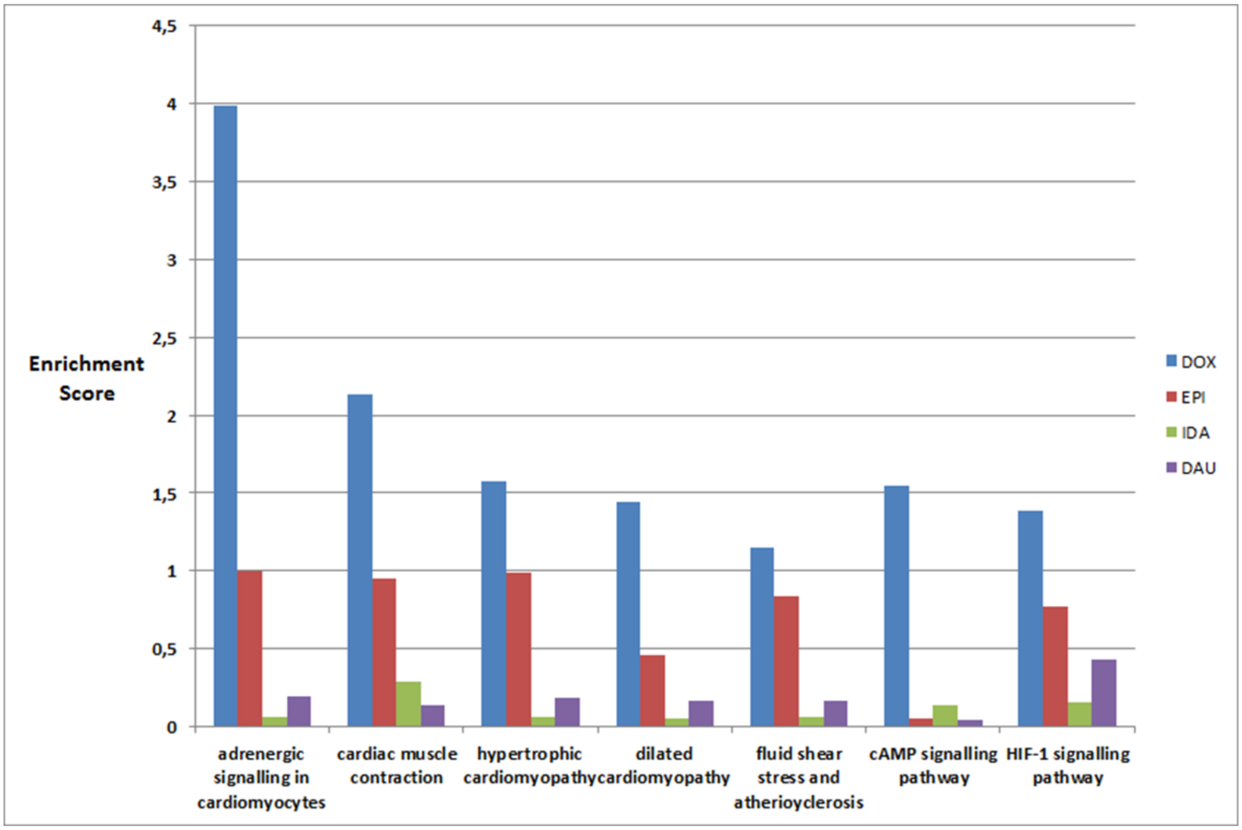

Supplementary Figure 4: Enrichment score (-log10(Q-value)) of heart disease pathways annotated by KEGG with the DEGs from DOX, EPI, IDA and DAU treatment vs. untreated rat hearts. Q-value is computed from a hypergeometric distribution and FDR correction.
